# Cost of integrating assisted partner services in HIV testing services in Kisumu and Homa Bay counties, Kenya: a microcosting study

**DOI:** 10.1186/s12913-022-07479-4

**Published:** 2022-01-14

**Authors:** Beatrice Wamuti, Monisha Sharma, Edward Kariithi, Harison Lagat, George Otieno, Rose Bosire, Sarah Masyuko, Mary Mugambi, Bryan J. Weiner, David A. Katz, Carey Farquhar, Carol Levin

**Affiliations:** 1 Department of Global Health, University of Washington, 325 9th Avenue, Box 359909, Seattle, WA 98104 USA; 2 PATH- Kenya, Kisumu, Kenya; 3 Kenya Medical Research Institute, Nairobi, Kenya; 4 Ministry of Health, Nairobi, Kenya; 5 Department of Epidemiology, University of Washington, Seattle, USA; 6 Department of Medicine, University of Washington, Seattle, USA

**Keywords:** Cost, Integration, HIV, Assisted partner services, Kenya, Microcosting

## Abstract

**Background:**

HIV assisted partner services (aPS), or provider notification and testing for sexual and injecting partners of people diagnosed with HIV, is shown to be safe, effective, and cost-effective and was scaled up within the national HIV testing services (HTS) program in Kenya in 2016. We estimated the costs of integrating aPS into routine HTS within an ongoing aPS scale-up project in western Kenya.

**Methods:**

We conducted microcosting using the payer perspective in 14 facilities offering aPS. Although aPS was offered to both males and females testing HIV-positive (index clients), we only collected data on female index clients and their male sex partners (MSP). We used activity-based costing to identify key aPS activities, inputs, resources, and estimated financial and economic costs of goods and services. We analyzed costs by start-up (August 2018), and recurrent costs one-year after aPS implementation (Kisumu: August 2019; Homa Bay: January 2020) and conducted time-and-motion observations of aPS activities. We estimated the incremental costs of aPS, average cost per MSP traced, tested, testing HIV-positive, and on antiretroviral therapy, cost shares, and costs disaggregated by facility.

**Results:**

Overall, the number of MSPs traced, tested, testing HIV-positive, and on antiretroviral therapy was 1027, 869, 370, and 272 respectively. Average unit costs per MSP traced, tested, testing HIV-positive, and on antiretroviral therapy were $34.54, $42.50, $108.71 and $152.28, respectively, which varied by county and facility client volume. The weighted average incremental cost of integrating aPS was $7,485.97 per facility per year, with recurrent costs accounting for approximately 90% of costs. The largest cost drivers were personnel (49%) and transport (13%). Providers spent approximately 25% of the HTS visit obtaining MSP contact information (HIV-negative clients: 13 out of 54 min; HIV-positive clients: 20 out of 96 min), while the median time spent per MSP traced on phone and in-person was 6 min and 2.5 hours, respectively.

**Conclusion:**

Average facility costs will increase when integrating aPS to HTS with incremental costs largely driven by personnel and transport. Strategies to efficiently utilize healthcare personnel will be critical for effective, affordable, and sustainable aPS.

**Supplementary Information:**

The online version contains supplementary material available at 10.1186/s12913-022-07479-4.

## Introduction

In Kenya, approximately 1.4 million people are living with HIV (PLWH), of whom an estimated 79.5% of individuals aged 15-64 years are aware of their status; with lower rates among men compared to women (72.6 vs 82.7%, respectively) [1]. The World Health Organization (WHO) recommends scaling up of assisted partner services to address this gap as part of a comprehensive package of testing and care for PLWH, particularly among men who have lower HIV testing rates and tend to start antiretroviral therapy later in the course of their illness compared to women [1, 2].

HIV partner services entails trained healthcare workers consenting individuals diagnosed with HIV (index clients) for names and contact information for all sex partners in the last three years to notify these partners of their potential exposure to HIV and linking them to testing and care services [2]. There are two main types of partner services. In client referral, providers encourage index clients to notify their sex partners of their potential exposure and encourage them to test for HIV. In assisted partner services (aPS), healthcare workers facilitate exposure notification after an agreed time if the index client does not notify their partner (contract referral), direct notification without the involvement of the index client (provider referral), or accompany and support index clients when they disclose their status and the potential HIV exposure to their partner (dual referral). Healthcare providers do not reveal the identity of the index to partners when they contact partners via phone calls requesting them to receive HIV testing services (HTS) at a facility, or travel to partners’ homes or workplaces to offer HIV testing [2].

Previous research and implementation projects from sub-Saharan Africa (SSA) demonstrate that aPS is safe, acceptable, and effective in increasing the number of newly testing partners and partners testing HIV-positive compared to client referral [3–10]. In addition, aPS has also been shown to be cost-effective and affordable [11]. A budget impact analysis (BIA) in Kisumu County, Kenya demonstrated that aPS is affordable but its impact was highly sensitive to the level of uptake [12].

Kenya’s Ministry of Health (MOH) is integrating aPS into the national HTS program, where integration is defined as the creation of linkages between a new intervention (aPS) and existing programs (HTS) to improve healthcare delivery [13]. Estimating costs of integrating aPS into routine HTS programs is crucial for budgetary planning. Further, understanding how aPS costs vary by location and client volume is important in resource allocation. We sought to estimate aPS costs to provide guidance to program planners and providers introduce and scale up aPS in their health systems.

We evaluated the cost of integrating aPS in the HTS program in Kisumu and Homa Bay counties in Kenya by estimating the incremental costs associated with integrating aPS into routine HTS programs, including start-up and recurrent costs. We estimated the average unit cost per male sex partner (MSP) traced, tested, testing HIV-positive, and on antiretroviral therapy (ART). We generated cost profiles of key inputs to identify the key drivers of aPS program costs. We also calculated disaggregated incremental costs, average unit costs per MSP, and median time spent on aPS by facility.

## Methods

### Parent project and intervention description

This microcosting analysis was conducted within in the aPS scale-up project (NIAID R01AI134130), a collaborative implementation science study between the Ministry of Health (MOH) National AIDS and STI Control Program (NASCOP), PATH-Kenya and University of Washington, conducted in 31 health facilities in Kisumu and Homa Bay counties in western Kenya [14]. Details of the aPS scale-up project and its implementation procedures have been published [14]. The overall goal of the project was to implement and evaluate the effectiveness of aPS when integrated within routine HTS, and assess implementation outcomes including acceptability, demand, integration, implementation fidelity, and costs.

The aPS scale-up project focused on evaluating aPS as a strategy for increasing HIV testing among men [14]. Though aPS is offered to both male and female clients receiving HTS in Kenya, this project deliberately targeted adolescent girls and women and their partners to address challenges in finding men who are difficult to mobilize through other interventions and may increase the burden of HIV through risky sexual behavior if not tested and treated Briefly, female clients testing HIV-positive at participating facilities (female index clients) received information on aPS and were screened for eligibility by healthcare workers. Those eligible were ≥15 years of age - those between 15 and 18 years were emancipated minors as per Kenya guidelines [15], newly diagnosed HIV-positive, at low risk of intimate partner violence (IPV), not pregnant, and had at least one sexual partner within the last 3 years. Participants were classified to have either low, moderate, or high IPV risk using the IPV screening tool adopted from the national APS guidelines [16]. Women at low risk of IPV did not fear IPV from their partner or had never experienced any form of IPV (emotional, physical, sexual). Pregnant women were excluded as they were considered a vulnerable population and were instead offered home-based couple counseling and testing.

Consenting female index clients were asked to provide names and contact information for all MSPs in the last three years, a process called partner elicitation. HTS providers contacted MSPs *via* phone and/or physical (in-person) tracing to notify them of their potential HIV exposure and offer HIV testing. MSPs testing HIV-positive were asked to enroll in aPS and provide contact information for their female sex partners, who were also followed up, notified of their exposure, and offered HTS. Female index clients and sex partners testing HIV-positive were encouraged to link to care and followed up at 6 weeks, 6 months, and 12 months to assess linkage to care, ART initiation, and HIV viral load suppression.

### Cost data collection

We estimated the incremental financial and economic costs of integrating aPS into the HTS program using a payer perspective [17] following principles outlined in the Global Health Cost Consortium Reference Case [18]. Financial costs represent actual expenditure on goods and services, while economic costs reflect the value of resources used to produce output. We used activity-based ingredients approach to identify key aPS activities, inputs, resource use, and associated prices and values of goods and services. We identified key activity cost centers and used microcosting methods to quantify and value inputs from each activity across facilities.

Cost data were collected during three field visits. In August 2018, we collected start-up costs from MOH NASCOP and PATH offices associated with one-time planning, training and awareness activities that took place before the project started. We then collected recurrent costs one year after study initiation from 14 facilities in Kisumu (*n* = 8) in August 2019, and then in Homa Bay (*n* = 6) in January 2020. Facilities were purposefully sampled based on location (county, urban/peri-urban/rural) and client volume (based on patient volumes receiving HTS at the facilities) after consultation with the site team.

We extracted data from project expense reports and MOH budgets, and obtained supplementary information on all key activities and resource use for the aPS integration. We also conducted semi-structured interviews with key health personnel at the MOH and PATH, as well as facility administrators to obtain information on time use and shared program costs (rent, personnel salaries, and supply prices from MOH sources) that were not available from expense and budget reports. We disaggregated costs by facility to evaluate variations in incremental costs and average unit cost per MSP.

We included health system costs incurred during provider elicitation of MSPs from female index clients, as well as for phone and physical tracing for MSPs. We excluded costs of eliciting and tracing female sex partners of HIV-positive MSPs as this was not the primary focus of the costing analysis. We included the costs of HIV testing and linking MSPs testing HIV-positive to care, but did not include the costs of ART since these costs are incurred under the national HIV care and treatment program, which is separate from the HTS/aPS program. We also excluded the cost of research activities not part of routine aPS delivery.

### Cost analysis

We differentiated between new aPS costs, and shared program costs to support the integration of aPS services into the existing HTS program. New costs were those related to aPS inputs and activities not conducted prior to aPS scale-up e.g., aPS microplanning meetings, initial trainings, sensitization, transport costs for physical tracing, communication costs for phone tracing, personnel (service delivery including partner elicitation, phone and physical tracing, exposure notification, HIV testing, and linkage to care) and aPS supervision. Shared program costs from the current HTS program were allocated based on the share of the activity or input used in aPS. These included the share of program costs for vehicles, equipment, overheads, HTS supplies, health facility administration, and refresher training.

We distinguished between fixed and variable costs. Fixed costs included overheads (e.g. building costs, water, and electricity), capital (vehicles, equipment), and non-service delivery personnel costs (i.e. health facility administration and aPS supervision). We allocated building space based on the proportion of time in the HTS visit taken up by aPS activities at each facility. Rental costs were estimated from MOH rates for government facilities or rental rates from nearby commercial properties. Capital costs were annualized over the expected useful life (assumed to be five years) using a 3% annual discount rate [18]. Similarly, start-up costs (microplanning, sensitization and training), which occurred once during the project, were treated as a type of fixed costs and annualized over five years using a 3% discount rate.

We estimated variable costs by measuring resource use across the 14 facilities. Personnel time was captured as a proportion of full-time work allocated to aPS. Salaries were converted into hourly wages based on the assumption that full-time employment was equivalent to 2080 hours/year. We estimated personnel time using time-and-motion observation for partner elicitation at the clinic, and MSP outreach by phone and physical tracing. To estimate personnel time cost, we multiplied the cost per minute (including both salary and benefits) by the median time spent on aPS activities including: 1) partner elicitation by the number of female index clients seen, 2) phone tracing by the number of MSPs traced on phone accounting for approximately 40% repeat calls, and 3) physical tracing multiplied by the number of MSPs traced physically accounting for 10% repeat physical tracing attempts. Based on facility data reports, we assumed that of the MSPs who were successfully traced, 70% were traced on phone and 30% by physical tracing. Estimates for the phone calls and physical tracing attempts were based on facility reports and staff opinion.

Phone call costs were estimated as a percentage of airtime assigned to the facility per year used to call MSPs elicited through aPS. Transport costs were estimated by multiplying the number of expected commutes per year, mainly through public transport, by the average cost of each commute. For supplies and commodity costs, we observed resource use during HIV testing and multiplied the relevant quantities by input costs obtained from program budgets or centralized price lists.

Cost data were collected and analyzed in templates designed in Microsoft Excel (Microsoft, Redmond, USA). We adjusted costs to 2019 currency and converted to US dollars (USD) using the 2019 average exchange rate (1 USD = KSh 101) [19]. Additional details about the costing methodology, including the Excel file used for the analysis, are available in the Supporting Information.

### Program volume

We used data collected by the implementation project staff to obtain the number of MSPs traced, tested, tested HIV-positive, and on ART over a one-year period as recorded in the MOH HTS facility registers. These data were compiled from April 1, 2019 to March 31, 2020 to capture costs at least one year prior to the COVID-19 pandemic.

### Cost metrics

For each facility, we first estimated incremental costs by summing the start-up and recurrent costs. We then calculated the average unit cost per MSP traced, tested, testing HIV-positive, and on ART by dividing the incremental costs by the number of MSPs traced, tested, testing HIV-positive, and on ART, respectively. Lastly, we estimated the weighted average incremental facility cost per year by weighting the annual incremental costs in our sample of 14 facilities by the number of MSPs in each facility. We also explored all cost metrics by facility to assess how client volume and location affect total incremental and average unit costs per MSP. We estimated cost shares by activity and input to explore how resources and activities were utilized within aPS.

### Scenario analysis

We estimated the costs of integrating aPS under two scenarios: 1) as-implemented, which replicates the current national HTS program where financial support is received from both government and external funding sources, and 2) MOH-only, in which, based on expert opinion from MOH and site staff, we excluded costs associated with international non-governmental organizations (NGO) i.e., we assumed that all HTS providers transitioned into the MOH human resource system, that only MOH staff would supervise aPS delivery, and that no international NGO overhead costs were incurred.

### Ethical approval

This study received ethical approval from the Kenyatta National Hospital Ethical and Scientific Review Committee (P465/052017) and the University of Washington Institutional Review Board (STUDY00002420). This study was conducted in accordance with the Declaration of Helsinki, and all study participants gave informed consent for enrolment and follow-up prior to study participation.

## Results

### Program volume

The total number of MSPs traced, tested, testing HIV-positive, and on ART across the 14 facilities as well as the number of clients reached through aPS over the one-year study period did not differ substantially by county (Table 1). These MSPs were elicited from 710 female index clients (Kisumu: 379 [53%], Homa Bay: 331 [47%]).

**Table 1:** Program volume overall and by county

Program volume	Kisumu	%*	Homa Bay	%*	Overall	%*
Male sex partners traced	1027	49%	1048	51%	2075	100%
Male sex partners tested	869	53%	763	47%	1632	100%
Male sex partners testing HIV-positive	370	55%	298	45%	668	100%
Male sex partners on ART	272	56%	215	44%	487	100%

* % Row percentage, *ART* antiretroviral therapy

### Personnel time associated with integrating aPS into HTS

Providers spent approximately 25% of an HTS visit with a newly diagnosed index client conducting partner elicitation i.e. obtaining MSP contact information (HIV-negative clients: 13 out of 54 min; HIV-positive clients: 20 out of 96 min) (Table 2). The median time spent per MSP traced on phone and in-person was 6 min and 2.5 hours, respectively. More time was spent on physical tracing in Kisumu compared to Homa Bay (174 min vs 125 min) while minimal differences were noted in median time spent on partner elicitation and phone tracing in both counties.

**Table 2:** Median time spent per client on aPS-related activities

	Median time spent on partner elicitation among HIV-negative female index clients receiving HIV testing services (per client)			Median time spent on partner elicitation among HIV-positive female index clients receiving HIV testing services (per client)				
Health facility	Partner elicitation (min)	Partner elicitation + HIV testing services (min)	%^a^	Partner elicitation (min)	Partner elicitation + HIV testing services (min)	%^a^	Median time spent phone tracing per client (min)	Median time spent physical tracing per client (min)
**Kisumu County**								
Sub-county hospital KH1	12	50	24%	27	107	25%	6	125
Sub-County hospital KL6	22	73	30%	27	110	27%	10	148
Health center KL5	3	16	19%	3	16	19%	5	163
Health Center KL4	13	50	26%	15	119	17%	4	185
Health Center KL7	8	36	22%	13	57	23%	6	153
Sub-county hospital KH3	12	84	14%	27	126	19%	13	202
Health center KL8	16	83	19%	34	120	25%	10	194
Health Center KH2	10	39	26%	10	39	26%	9	307
**Median (Kisumu)**	**12**	**50**	**24%**	**21**	**109**	**19%**	**6**	**174**
**Homa Bay County**								
Health Center HH2	17	66	26%	37	106	31%	5	114
Sub-county hospital HH3	15	60	25%	20	92	23%	8	97
Dispensary HL6	9	53	17%	19	81	21%	3	155
County hospital HH1	17	56	31%	30	88	32%	9	137
Sub-county hospital HL5	15	59	25%	20	73	26%	8	114
Dispensary HL4	9	53	17%	14	75	18%	2	170
**Median (Homa Bay)**	**15**	**57**	**26%**	**20**	**84**	**23%**	**6**	**125**
**Overall median**	**13**	**54**	**25%**	**20**	**96**	**21%**	**6**	**150**
**Range (Min-Max)**	**3-22**	**16-84**	**14-31%**	**3-37**	**16-126**	**17-32%**	**2-13**	**97-307**

^a^*% Percentage*

### Incremental costs of integrating aPS

The weighted average incremental cost of aPS was $7,485.97 per facility per year (as-implemented), with recurrent costs accounting for approximately 90% of costs (Table 3). Personnel (49%) accounted for the largest share of costs followed by transport (13%). The proportion of costs appropriated to different categories was similar in both counties apart from personnel costs, which accounted for 54% of incremental costs in Kisumu, compared to just 35% in Homa Bay, mainly due to differences in personnel time spent on physical tracing.

**Table 3:** Annual average weighted incremental facility cost – As implemented vs MOH-only scenario (2019 USD)

	As implemented						MOH-only scenario					
	Kisumu	%	Homa Bay	%	Overall	%	Kisumu	%	Homa Bay	%	Overall	%
**Start-up costs**												
Microplanning	$483	5%	$432	9%	$459	6%	$483	7%	$432	12%	$459	8%
Initial Training	$466	5%	$416	9%	$443	6%	$466	7%	$416	11%	$443	8%
Sensitization	$90	1%	$17	0%	$56	1%	$90	1%	$17	0%	$56	1%
**Sub-total**	**$1,040**	**10%**	**$865**	**18%**	**$958**	**13%**	**$1,040**	**15%**	**$865**	**23%**	**$958**	**17%**
**Recurrent costs**												
Personnel	$5,407	54%	$1,632	35%	$3,642	49%	$3,213	46%	$1,215	33%	$2,279	41%
HTS supplies	$573	6%	$500	11%	$539	7%	$573	8%	$500	13%	$539	10%
Equipment	$28	0%	$29	1%	$28	0%	$28	0%	$29	1%	$28	1%
Vehicles	$215	2%	$0	0%	$114	2%	$215	3%	$0	0%	$114	2%
Transport	$1,260	13%	$695	15%	$996	13%	$1,260	18%	$695	19%	$996	18%
Communication	$165	2%	$40	1%	$107	1%	$165	2%	$40	1%	$107	2%
Overhead	$758	8%	$580	12%	$675	9%	$72	1%	$18	0%	$46	1%
Refresher training	$489	5%	$355	8%	$426	6%	$489	7%	$355	10%	$426	8%
**Sub-total**	**$8,896**	**90%**	**$3,831**	**82%**	**$6,528**	**87%**	**$6,015**	**85%**	**$2,851**	**77%**	**$4,536**	**83%**
**Summary**	**$9,935**	**100%**	**$4,696**	**100%**	**$7,486**	**100%**	**$7,055**	**100%**	**$3,717**	**100%**	**$5,494**	**100%**

* % Percent of total costs

After excluding international NGO costs (MOH-only scenario), the weighted average incremental cost of integrating aPS was $5,494.06 per facility per year. The reduction in costs was due to a decrease in personnel costs specifically aPS supervision costs (from 49 to 41%) and overheads especially rental leases for NGO offices (from 9% to 1%) (Table 3).

### Average unit costs per MSP

The average unit cost per MSP traced, tested, testing HIV-positive, and on ART (as-implemented) was $34.54, $42.50, $108.71, and $152.28, respectively (Table 4). These costs were much higher in Kisumu compared to Homa Bay County due to differences in personnel time spent on physical tracing. In the MOH-only scenario, the average unit costs per MSP traced, tested, testing HIV-positive, and on ART were $25.55, $31.59, $81.42, and $114.17, respectively, largely due to lower personnel and international NGO overhead costs (Table 4).

**Table 4:** Average unit cost per MSP – As implemented vs MOH-only scenario (2019 USD)

	As implemented			MOH-only scenario		
Average unit cost	Kisumu	Homa Bay	Overall	Kisumu	Homa Bay	Overall
Cost per male sex partner traced	$46.56	$20.86	$34.54	$33.42	$16.60	$25.55
Cost per male sex partner tested	$54.96	$28.30	$42.50	$39.51	$22.57	$31.59
Cost per male sex partner testing HIV-positive	$135.29	$78.44	$108.71	$97.79	$62.78	$81.42
Cost per male sex partner on ART	$183.02	$117.26	$152.28	$131.82	$94.07	$114.17

* ART Antiretroviral therapy

### Direct aPS activity costs and shared program costs

**Fig. 1:**
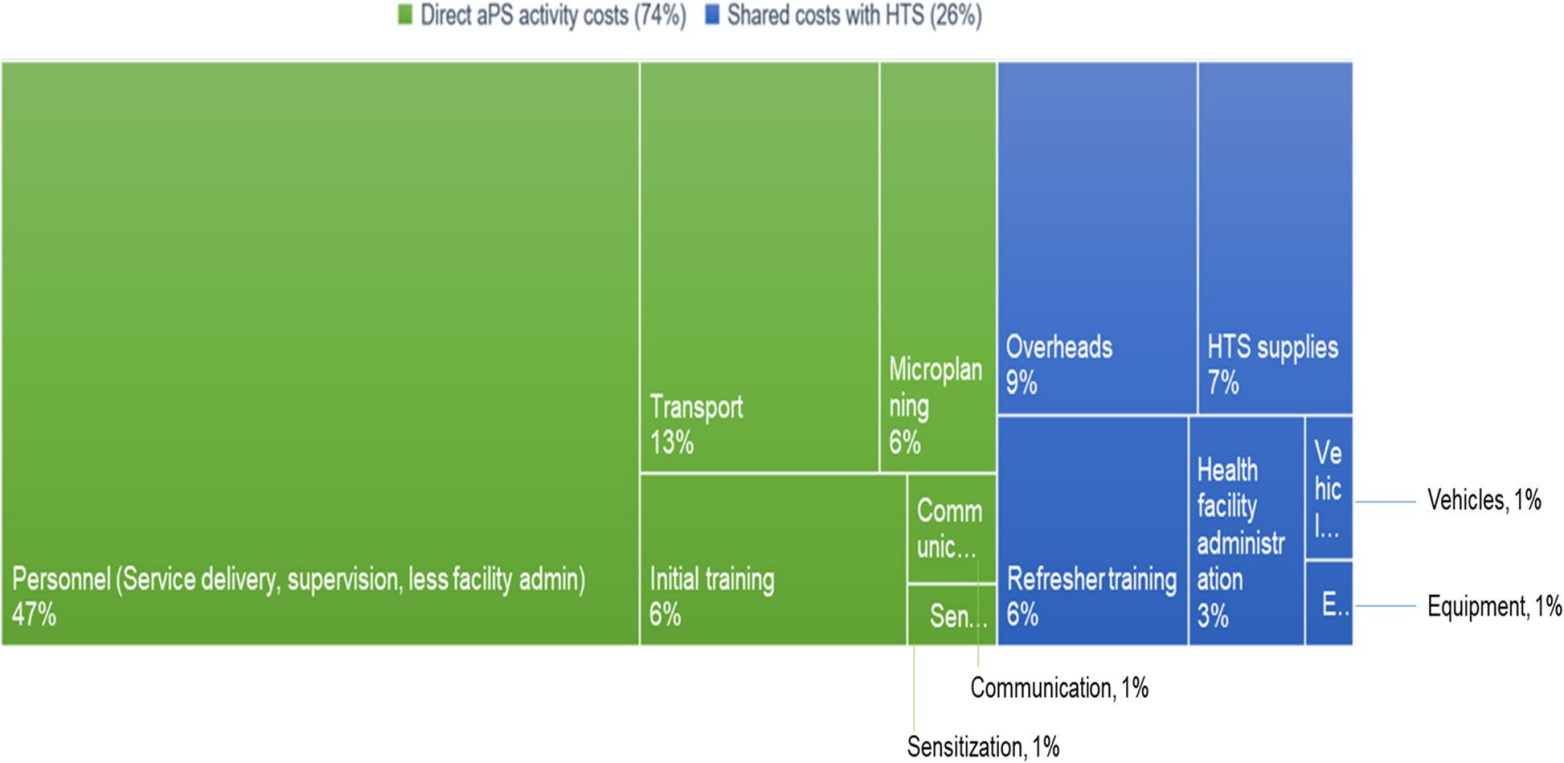
Direct aPS activity costs compared to shared program costs of integrating aPS into HTS

APS program specific delivery costs accounted for 74% of the incremental costs of aPS, while the remainder were shared costs related to overhead, training, administration, and supply costs of the existing HTS program (Fig. 1). For aPS delivery activities, the largest cost drivers were personnel and transport in both counties. However, direct aPS delivery costs were higher in Kisumu compared to Homa Bay County (77% versus 66%) mainly due to more personnel time spent on physical tracing.

### Fixed and variable costs

**Fig. 2:**
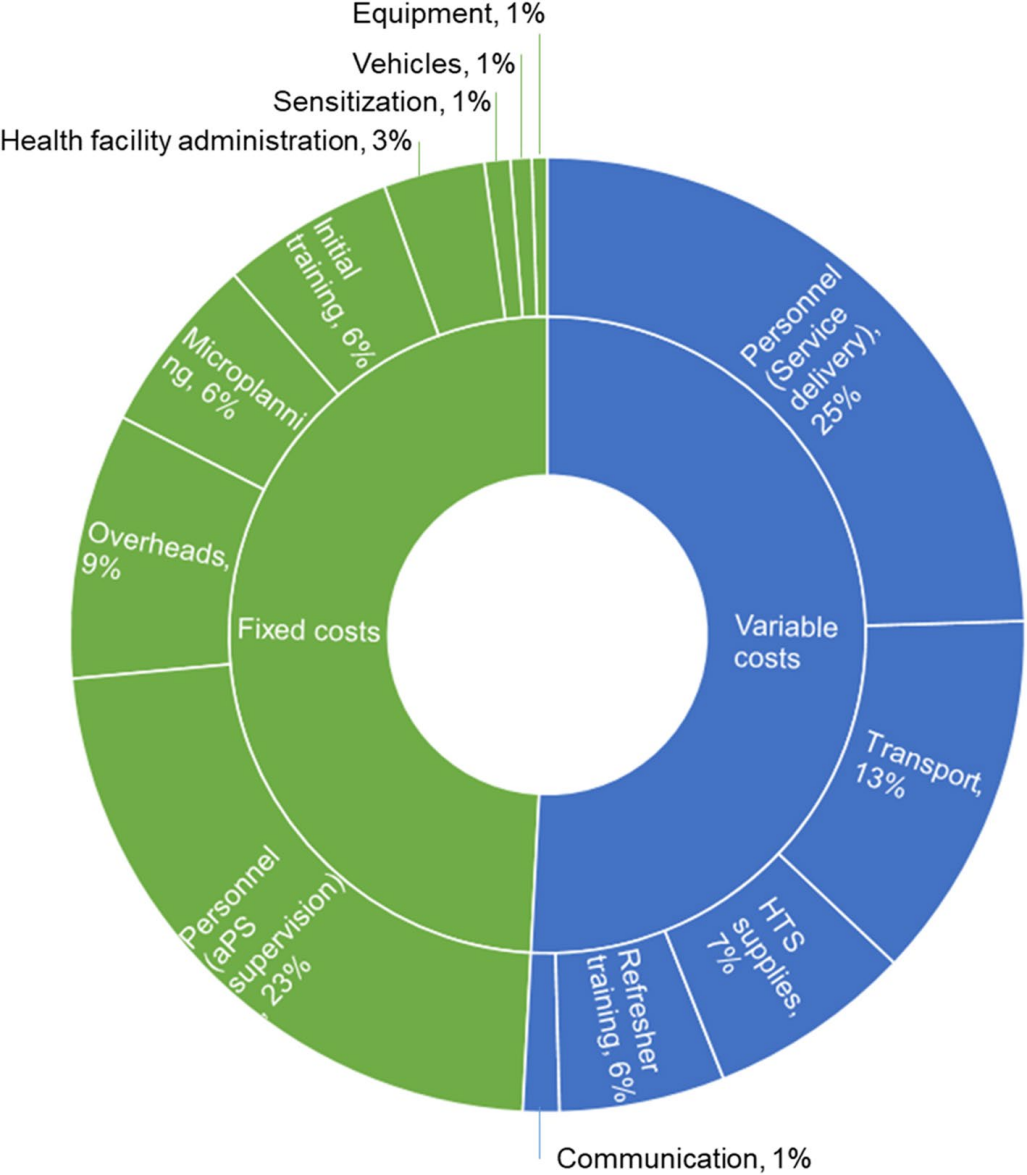
Proportion of total incremental costs by input type for aPS integration.

Overall, costs were evenly split into fixed to variable costs, with no difference in shares between the two counties (Fig. 2).

### Costs by facility

**Fig. 3:**
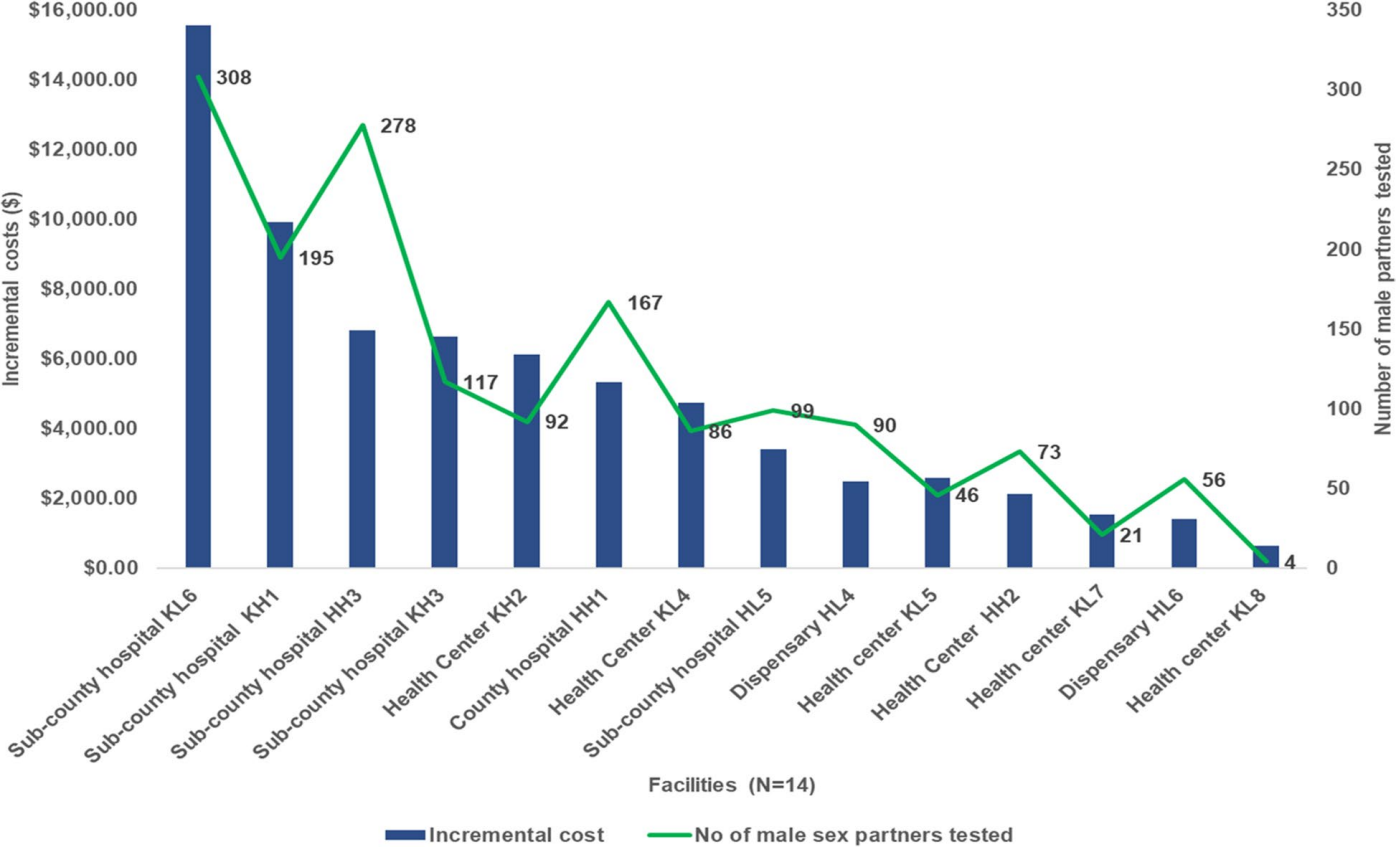
Annual incremental cost of aPS in 14 facilities in Kisumu and Homa Bay County (As-implemented), 2019 USD

**Fig. 4:**
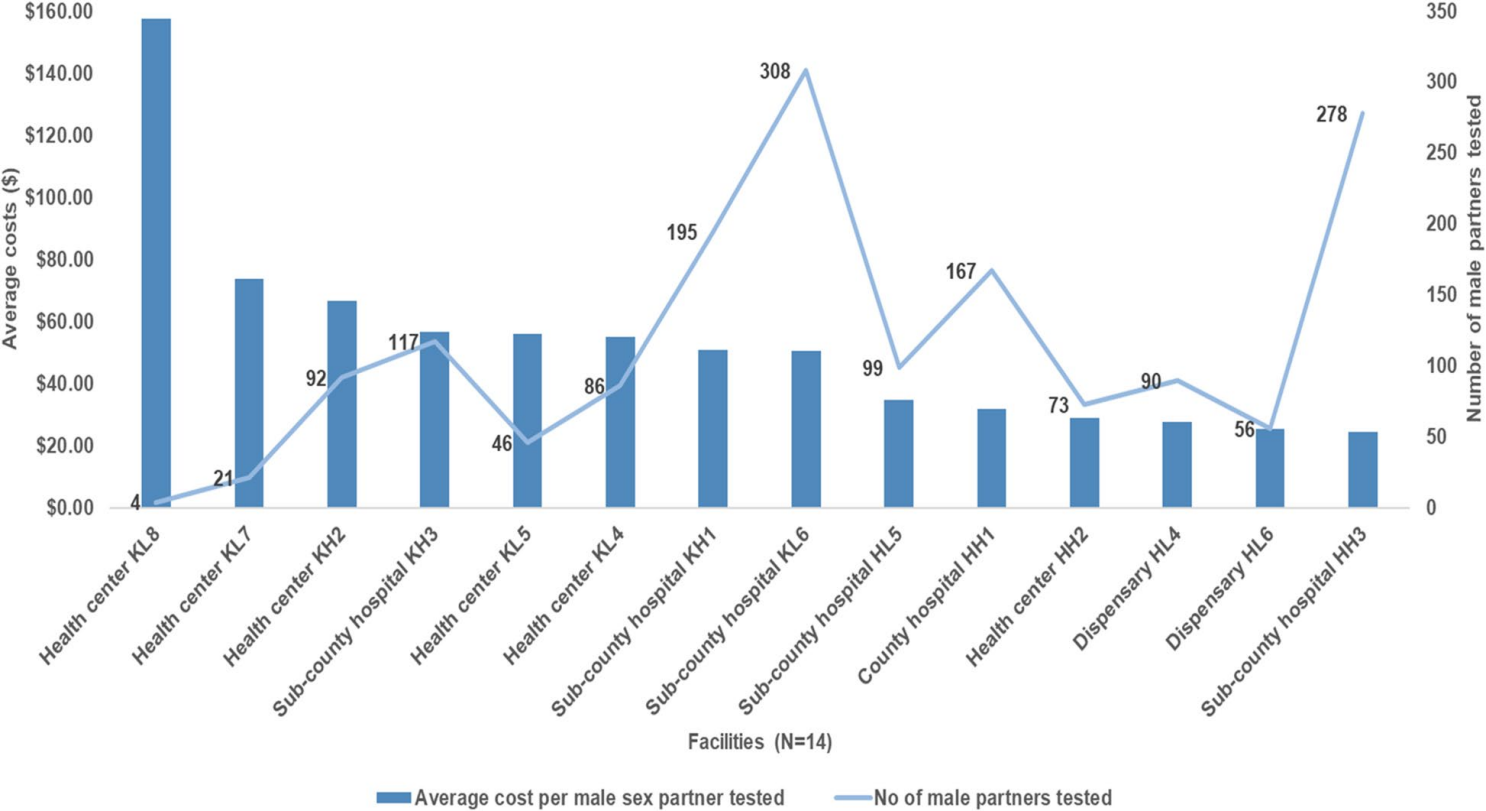
Average cost of aPS per male sex partner tested in 14 health facilities in Kisumu and Homa Bay countries (As-implemented), 2019 USD

When exploring costs and outputs by facility, the total incremental costs varied substantially, from $630.06 to $15,572.24 per facility per year, while the average unit cost per MSP testing HIV-positive ranged from $24.56 to $157.52. Generally, the highest incremental costs and lowest average unit costs per MSP were observed in larger volume facilities, particularly county and sub-county hospitals, which had higher client volumes (Figs. 3 and 4).

## Discussion

In this microcosting analysis, we estimate that integrating aPS into HIV testing services in Kisumu and Homa Bay county facilities targeting MSPs to female index clients would increase overall HTS cost by approximately $7,485 per facility per year, mostly driven by recurrent costs (especially personnel and transport). The estimated annual budget at a high-volume county referral hospital in Kisumu is $720,000, and facility-level aPS integration accounts for approximately 1% of the budget; therefore, aPS may be affordable in such a large facility, but may be less so in lower volume healthcare facilities with smaller budgets. We estimated higher incremental and lower average unit costs per MSP in larger volume facilities, which is expected as aPS implementation is more costly per client in low volume clinics since overhead costs are spread over fewer clients. This may highlight the need to prioritize resources towards higher volume facilities that can then support aPS in multiple low volume facilities within their vicinity to increase efficiency; however, this may lead to missed opportunities for aPS in lower volume facilities. The majority of the integration costs were direct aPS costs related to personnel (service delivery, aPS supervision) and transport, with approximately 25% of aPS integration costs shared with the existing HTS program (overhead, HTS supplies, refresher training), indicating the proportion of pre-existing HTS resources that would be needed to support aPS. Based on staff opinion, this 26% did not seem to overwhelm the facility healthcare system; they reported that aPS led to more optimal use of facility resources as more clients could be targeted to receive not only HTS, but also other available healthcare services (Personal communication).

Across facilities, personnel made up the largest portion of total costs (49%), followed by transport (13%). Kisumu County had higher personnel costs than Homa Bay as HTS providers spent more time physically tracing partners in Kisumu - where MSPs were harder to trace since they more frequently changed their places of work and residence, compared to those in Homa Bay - a rural county where clients change residence less often. This finding is consistent with qualitative results on aPS from our study [20]. The higher personnel and transport costs for MSP tracing highlight the importance of identifying methods to increase efficiency, e.g. batching visits, reducing distance traveled by HTS providers, utilizing community health workers or volunteers (CHWs, CHVs) to support physical tracing, incentivizing partners to come to the clinic for testing, and improving their mobile phone access.

This study complements previous estimates by providing an accurate estimate of the resources needed to scale up aPS as part of a routine HTS program in Kenya. Our unit cost estimates were slightly lower to prior studies that estimated aPS implementation cost as part of randomized control trials (RCT) settings [4, 12]. In an aPS RCT targeting both male and female sex partners in Kenya, estimated costs per partner tested were $48–55 using a program scenario with highly trained HTS providers i.e. health advisors [11]. In the same RCT, the cost of aPS ranged between $44.75 and $53.07 per client for nurse-based testing, and between $32.04 and $33.72 per client for CHW-based testing [12]. Our estimated average unit cost per MSP tested using regular HTS providers was slightly lower (as-implemented: $42.50, MOH-only scenario: $31.59), though our cost estimates focused on MSPs only and not both male and female partners. Relative to other HIV prevention strategies e.g., HIV self-testing (~$10 per client tested), voluntary medical male circumcision ($66 per procedure), and prevention of mother to child transmission ($79 per client tested), aPS is likely an affordable, high-yield HIV prevention intervention that can be used to target relatively hard-to-reach populations such as men [21–25]. Our team is currently evaluating a combined aPS and HIV self-testing strategy and its cost implications, the results of which will improve our understanding of combined HIV prevention strategies.

Compared to previous estimates, our unit costs may have been lower as we utilized HTS providers who are paid lower than health advisors or nurses used in prior aPS costing studies [11, 12]. Though CHWs – who earn substantially lower salaries - are not yet approved to offer HTS and aPS in Kenya - task-shifting scenarios using this cadre have been shown to lower costs per partner tested and could potentially be used to offer aPS once approved [12, 26]. Whether integrating aPS is sustainable will depend on availability of funds, priorities of the MOH and external funders, and willingness to scale-up and sustain aPS in the long-term. With declining funds and potential transition of HIV management from external funders to the government, key policy makers at national and county levels may have to adjust their budgets accordingly to ensure longevity of this intervention.

Similar to other aPS costing studies, personnel was a major cost driver accounting for 40-70% of total costs [11, 12]. In an attempt to reduce personnel costs, the aPS scale-up project began using a hub-and-spoke model [27] in which HTS providers stationed at high volume facilities supported several lower volume facilities. While this occurred after our cost data collection had concluded, a hub-and-spoke strategy has the potential to improve efficiency given the lower average unit costs per MSP in high-volume facilities [28]. Other strategies to improve efficiency include community sensitization on aPS to increase awareness and encourage partners to uptake HTS at a facility, reducing personnel time and costs for phone and physical tracing [28]. Ministries of health may also consider transferring physical tracing to HTS providers stationed at health facilities closest to partners. However, this approach needs to be carefully reviewed in conjunction with the index clients due to concerns on privacy and confidentiality, and risks of intimate partner violence [5, 29].

There are several limitations in our study. By focusing only on MSPs, we were not able to estimate the cost of aPS for female sex partners. However, we anticipate most unit costs would be similar, apart from partner elicitation costs and physical tracing costs that may vary by target group. Secondly, our study focused on only 14 facilities in Kisumu and Homa Bay counties, which have the highest HIV prevalence in Kenya (>15%) and might not be representative of other counties or settings or easily generalizable [1]. However, costs in lower volume facilities in our study might be comparable to those in lower prevalence counties in Kenya. Third, we utilized a payer perspective and do not account for costs incurred or saved by participants receiving aPS in clinics or HTS in the community. Providing aPS in the community can reduce individual costs of time and transport to the facility; therefore, societal costs would be lower. Finally, we did not include ART costs in our analysis since these are covered by the HIV care and treatment program which is separate from the HIV/aPS program. In Kenya, ART costs are estimated to be between $70 - $215 per person per year and these costs would need to be factored in for the additional HIV-positive clients reached through aPS [30].

Our study has several strengths. We evaluated the cost of integrating aPS into HTS programs within real-world settings, giving a realistic estimate of the implementation costs. We disaggregated costs to identify variations across facilities and propose strategies to improve cost efficiency. Finally, through detailed time-and-motion studies, we estimated the median time and cost of providing aPS during HTS, which will facilitate resource planning particularly human resource allocation and transport reimbursements.

## Conclusion

This study contributes to the growing literature on the cost of integrating aPS, and while average facility costs are expected to increase when integrating aPS to HTS, this increase is within the expected range [4, 12, 31]. As aPS is scaled-up, especially in resource-limited settings and as funding allocated to HTS shifts, additional research on cost-efficient strategies optimizing resource allocation during aPS is critically important.

## Supplementary Information

**Additional file 1.**

## Data Availability

The datasets used and/or analysed during the current study are available from the corresponding author on reasonable request.
